# Skin maculae, chronic diarrhea, cachexia, and splenomegaly—Late presentation of the first autochthonous case of visceral leishmaniasis in Tanzania

**DOI:** 10.1371/journal.pntd.0008925

**Published:** 2021-01-14

**Authors:** Oliver Henke, Priscus John Mapendo, Alex Mremi, Lilian Gasper Mmbaga, Angela Elisha Pallangyo, Thomas Harbaum, Elifuraha Mkwizu

**Affiliations:** 1 Cancer Care Centre, Kilimanjaro Christian Medical Centre, Moshi, United Republic of Tanzania; 2 Department of Pathology, Kilimanjaro Christian Medical Centre, Moshi, United Republic of Tanzania; 3 Joint Medical Service of the German Armed Forces, Department of Public Health, Koblenz, Germany; US Food and Drug Administration, UNITED STATES

## Abstract

A 20-year-old man from Simanjiro district in northern Tanzania presented with a 3-year history of splenomegaly, fatigue, cachexia, skin maculae, and recent onset of watery diarrhea at Kilimanjaro Christian Medical Centre (KCMC) in Northern Tanzania. Due to laboratory findings of pancytopenia, diagnostic workup included bone marrow aspiration cytology and biopsy. Although the rapid test (IT LEISH, rK39 RDT) was negative, blood smear showed amastigote forms of leishmaniasis in macrophages. Repeat bone marrow aspiration and PCR eventually confirmed visceral leishmaniasis (VL). The patient denied travel to known endemic areas of VL. Treatment was initiated with Amphotericin B, but the patient died on the fourth day of treatment from respiratory insufficiency. An autopsy revealed massive organ manifestations of VL. This is the first reported autochthonous case of VL in Tanzania. Clark and colleagues detected the vector *Phlebotomus martini* in Northern Tanzania in 2013, in a region bordering the district of our patient. The negative rapid test draws attention to the fact that sensitivity and specificity were found to be low in East African VL patients as displayed earlier by a Kenyan study. Therefore, tissue samples (spleen or bone marrow) remain necessary for diagnosis. The variety of symptoms in this presented case was remarkable, including the occurrence of post-kala-azar dermal leishmaniasis (PKDL) and VL at the same time. This has been described in East African VL cases before as well as the occurrence of chronic diarrhea. An elongated undiagnosed period likely led to a mixed clinical picture that included hepato-splenomegaly, PKDL, cachexia, and diarrhea.

## Case presentation

We describe a 20-year-old male from the Mbuyuni area of northern Tanzania who presented to Kilimanjaro Christian Medical Center (KCMC) in January 2020 with weight loss, splenomegaly, severe cachexia, and watery diarrhea. However, the leading symptom on presentation was watery diarrhea for 3 weeks.

The patient was accompanied by his father who reported that his son suffered from cachexia starting age 17. Prior to age 17, his medical history was unremarkable. His parents and 7 siblings are all healthy.

At the time of initial symptom onset, the patent lived in the Simanjiro district for cattle herding, while the rest of his family members lived in the suburban area of Arusha. He reported several insect bites during his period of working as a cattle herder. His symptoms gradually worsened with increasing weight loss and splenomegaly and as well as skin rashes and maculae in his face. After a year, he sought treatment from traditional healers. After another year of no improvement from consultation of the traditional healer, he presented to an outside hospital in the Arusha region (unknown diagnostic and treatment approach). Eventually, he was admitted at KCMC for further diagnostics.

On admission to KCMC, the patient’s primary complaints were abdominal pain and swelling. The laboratory tests on admission revealed white blood count of 1.51 × 10^9^/L (neutrophils 37.3%, lymphocytes 58.4%, monocytes 3.7%, eosinophils 0.4%, and basophils 0.2%), red blood cell count 2.21 × 10^12^/L, hemoglobin 4.9 g/dL, thrombocytes 56 × 10^9^/L, creatinine 41 μmol/L, aspartate aminotransferase (AST) 12.9 U/L, alanine aminotransferase (ALT) 7.6 U/L, sodium 124.6 mmol/L, potassium 2.9 mmol/L, and chloride 106 mmol/L. HIV test result is negative. The sputum polymerase chain reaction (PCR) for tuberculosis (GenXpert, Cepheid, Sunnyvale, California, United States of America) was negative; a chest X-ray investigation was normal; and ultrasound of the abdomen revealed hepatosplenomegaly.

**Fig 1:**
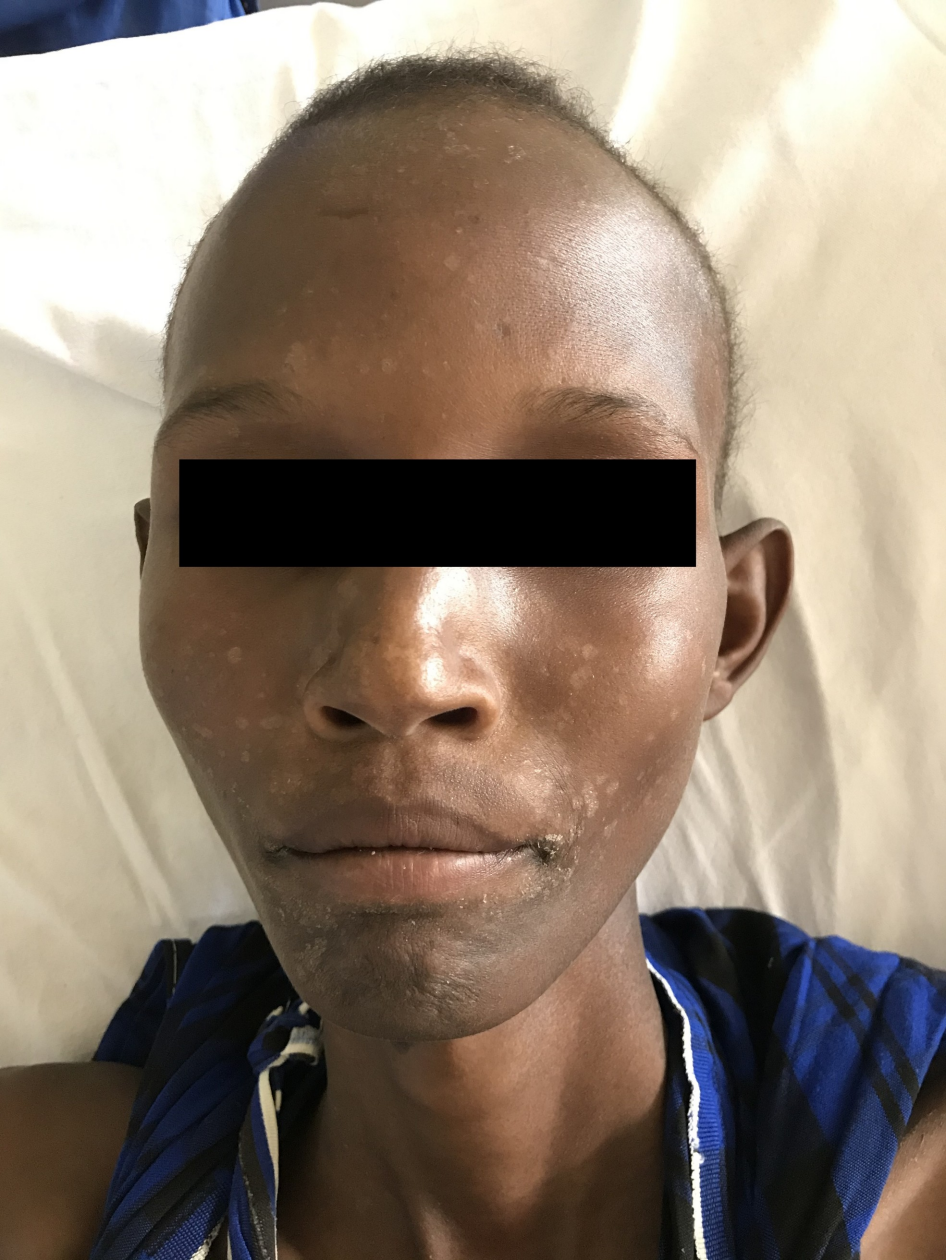
Hypopigmented spots perioral as a feature of post-kala-azar dermal leishmaniasis.

On physical exam, he appeared pale and was wasted with a body weight of 39 kg and a height of 172 cm. On abdominal exam, his spleen was enlarged 18 cm below costal margin; the liver was enlarged 3 cm below costal margin; there were traditional healer marks throughout the abdomen; there were no palpable lymph nodes; and the lungs and heart were normal. His skin exam demonstrated hypopigmented maculae scattered on his face with accentuation in the perioral region (Fig 1) and skin-colored maculae on his trunk and arms. The latter occurred a few months before presentation in the hospital. Alopecia areata was also noted.

His travel history includes a 3-month stay in Mombasa (Kenya), approximately 1 year prior to presentation, where he worked as a merchant. Travels to other areas in Kenya or other countries, especially to endemic VL areas, has been denied by the patient as well as travels within Tanzania.

**Fig 2:**
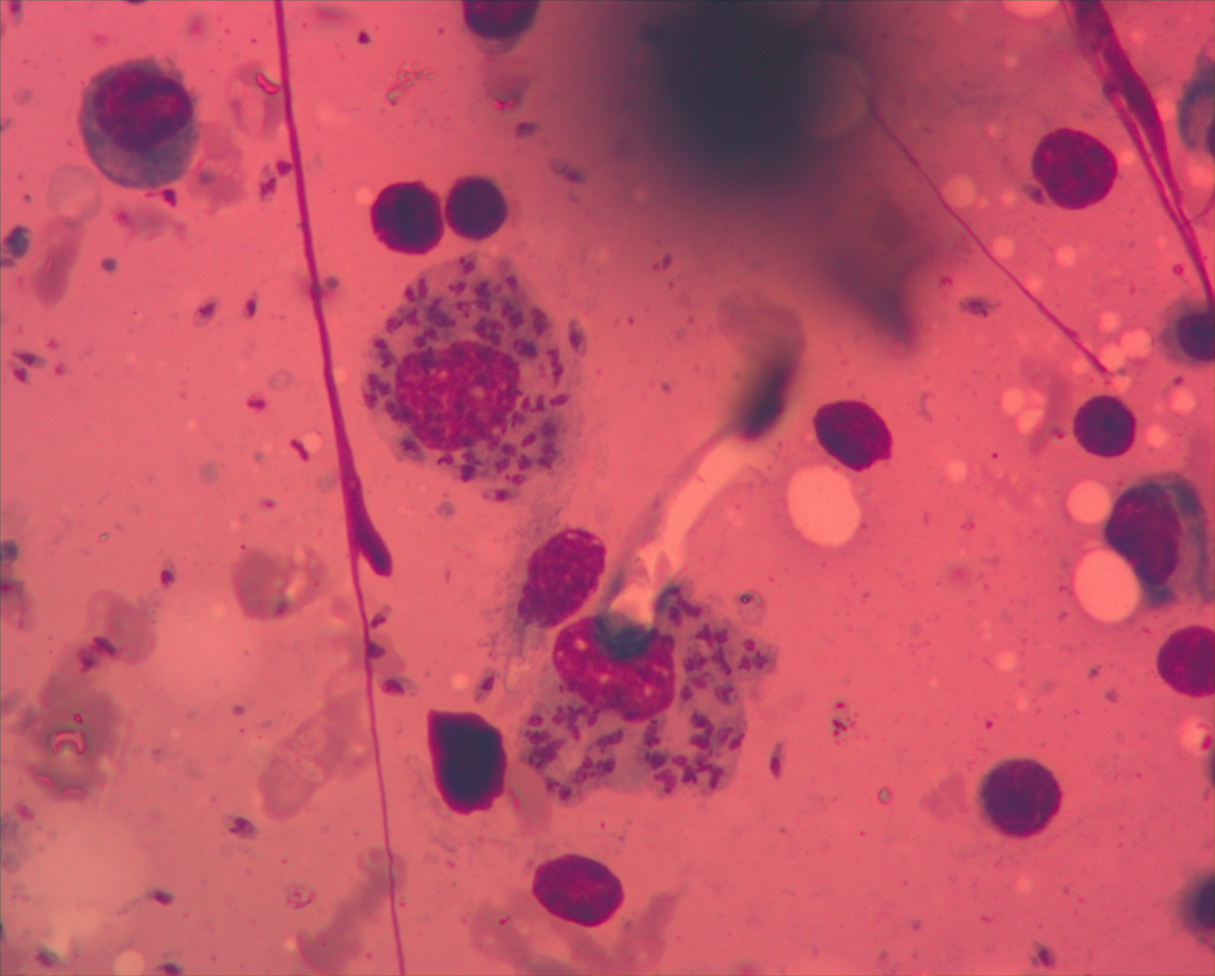
Bone marrow aspirate: Amastigotes in macrophages (Wright staining; ×1000 magnification).

**Fig 3:**
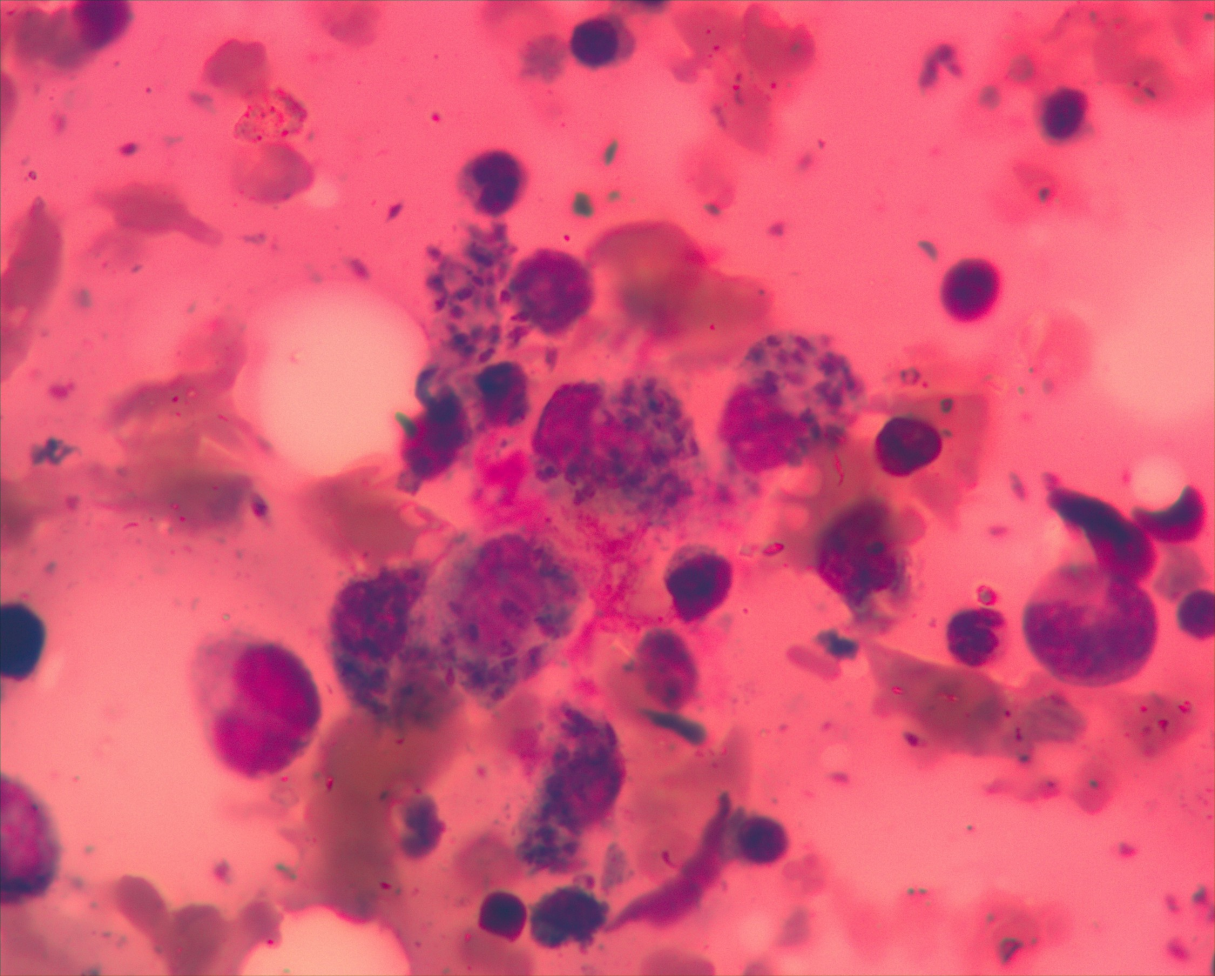
Bone marrow aspirate: Amastigotes in macrophages (Wright staining; ×1000 magnification).

Due to pancytopenia, the patient was referred to the Hematology section of the Cancer Care Centre at KCMC, where we performed bone marrow aspirate and biopsy, revealing *Leishmania* amastigotes in macrophages (Figs 2 and 3). The rapid test for *Leishmania* antibody detection (IT LEISH) from the peripheral blood and the bone marrow aspirate remained negative.

After discussion with senior experts in tropical diseases, we decided to reperform bone marrow aspiration for PCR in a reference laboratory, which found the aspirate positive for *Leishmania* species DNA.

**Fig 4:**
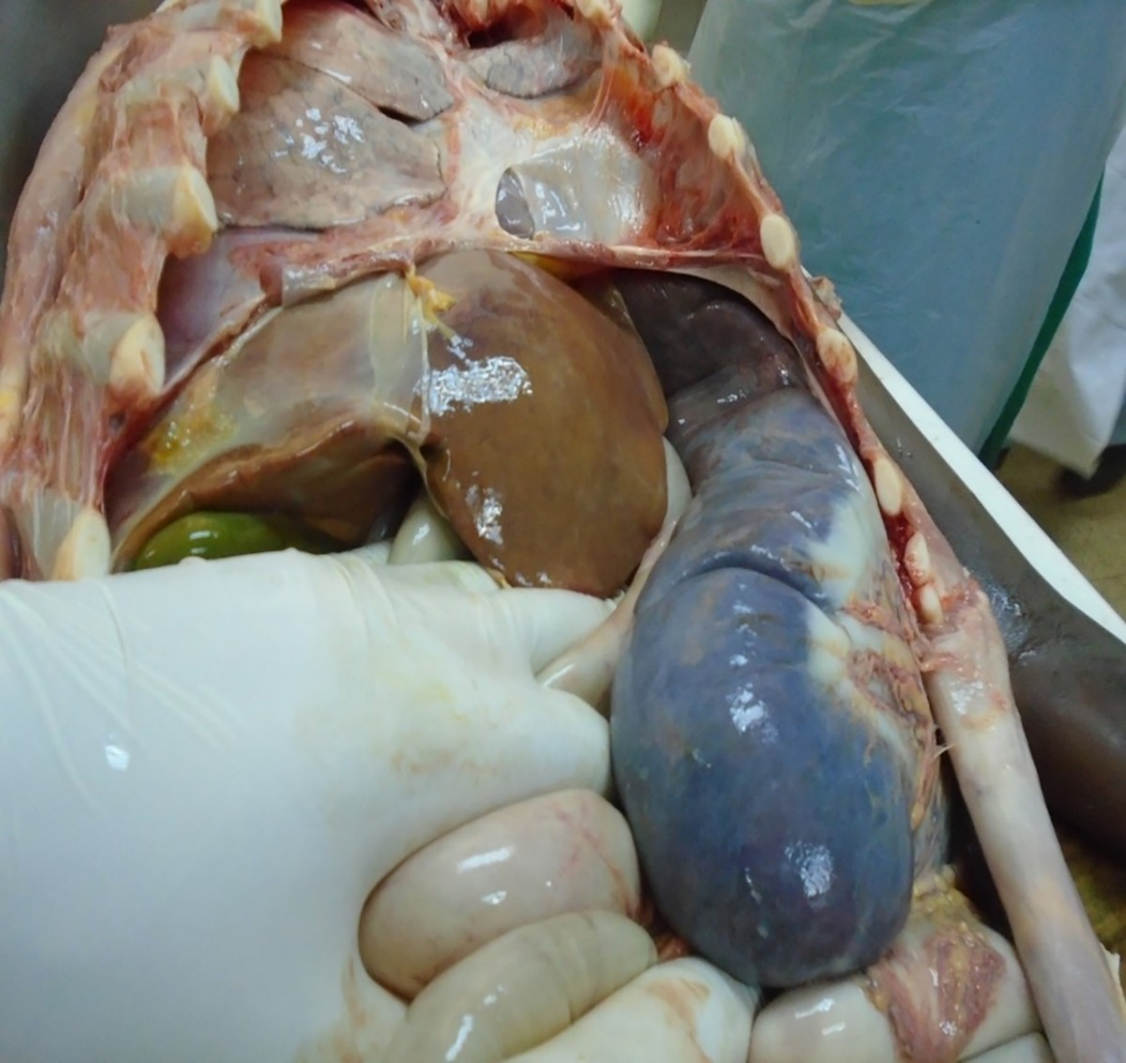
Postmortem finding: massive splenomegaly and hepatomegaly.

**Fig 5:**
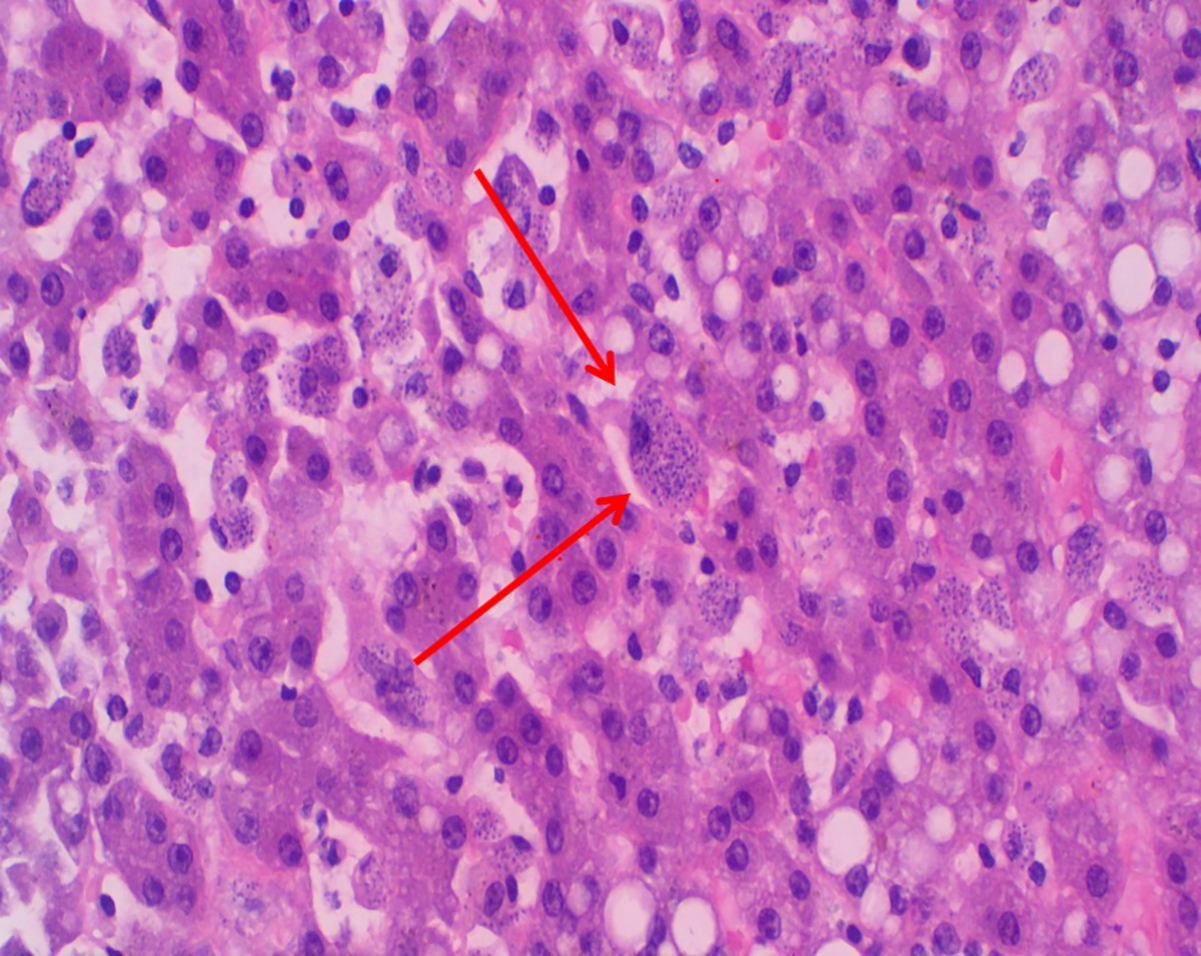
Histopathology of liver showing abundant intra- and extracellular *Leishmania* amastigotes (H&E staining; ×200 magnification).

**Fig 6:**
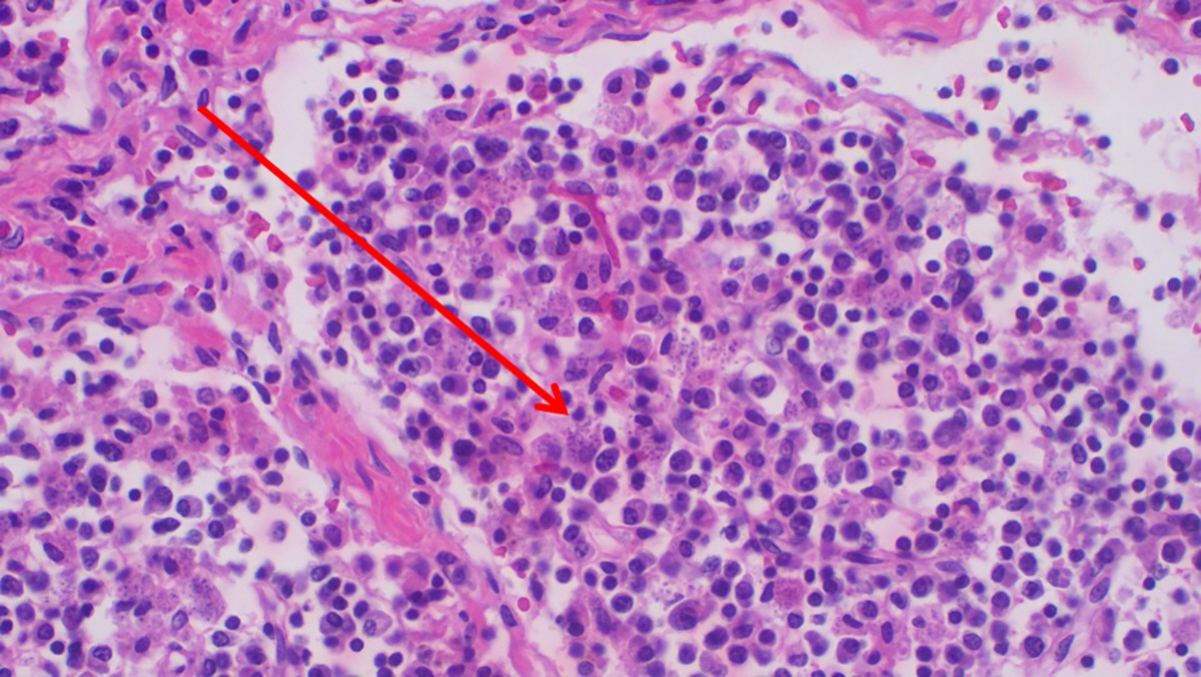
Histopathology of spleen showing abundant intra- and extracellular *Leishmania* amastigotes (H&E staining; ×200 magnification).

**Fig 7:**
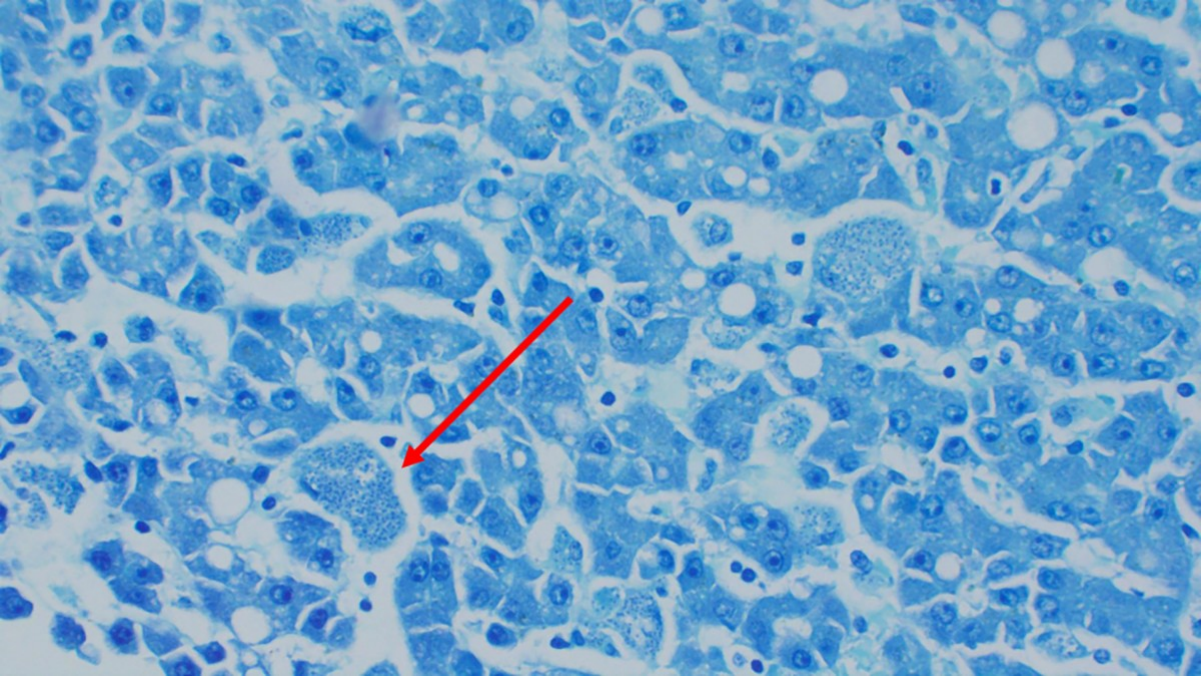
Histopathology of liver showing intracellular amastigotes (Giemsa staining; ×200 magnification).

Due to the unavailability of liposomal Amphotericin, we initiated treatment with Amphotericin B (1 mg/kg IV). Unfortunately, the patient passed away on the fourth day of treatment due to sudden onset of respiratory insufficiency. Beside evidence of massive organ manifestations of VL (Figs 4–7), the autopsy revealed pneumonia (Table 1).

**Table 1:** Timeline of the hospital stay at KCMC.

22 January 2020	First admission with diarrhea, weight loss, and splenomegaly. Abdominal ultrasound: hepatosplenomegaly; Full blood count: pancytopenia’; peripheral blood smear: no signs of parasitemia. Chest X-ray: normal. Urine dip stick: cystitis. Antibiotic treatment initiated. Decision for referral to Hematology section for bone marrow biopsy.
4 February 2020	Bone marrow aspiration and biopsy performed; intracellular amastigotes seen. rK39 (peripheral blood) rapid test negative. VL and toxoplasmosis considered as diagnosis. Decision for supportive treatment (blood transfusion and antibiotic treatment) and discharge of patient. Awaiting results of pathology report and discussion of bone marrow aspirate slides with consultant in infectiology and tropical medicine (VL likely diagnosis despite negative rK39).
28 February 2020	Second bone marrow aspirate for rK39 (bone marrow aspirate) and PCR (via private laboratory; sent to South Africa) as ambulatory patient. Rapid test was again negative.
19 March 2020	PCR result is positive, pathology report describes amastigotes as well. Readmission of patient and correction of hypokalemia (diarrhea) before treatment initiation.
25 March 2020	Treatment start with Amphotericin B.
29 March 2020	Sudden onset difficulty in breathing, chest X-ray with infiltrates; initiation of antibiotic treatment. The patient eventually died the same night.

KCMC, Kilimanjaro Christian Medical Centre; VL, visceral leishmaniasis.

## Case discussion

VL is endemic in the East African countries of Sudan, South Sudan, Ethiopia, Kenya, Uganda, and Somalia with an annual incidence between 29,400 and 56,700 cases, accounting for approximately 15% of the global cases [1]. The largest VL epidemic ever recorded took place during the1980s in Sudan, where an estimated 100,000 people died. [2] Another common risk factor in East Africa contributing to the rise in VL cases is a high prevalence of HIV. [2]

This case is remarkable as it is the first reported autochthonous case of VL in Tanzania [3]. Clark and colleagues reported about the occurrence of the vector *Phlebotomus martini* in northern Tanzania in 2013 [4]. The sand fly species was found in 5 sites within the Kilimanjaro and Arusha regions, both bordering Simanjiro district, where our patient resided. In the Arusha region, *P*. *martini* account for 18% of all sand fly species [4] and are described to be especially prevalent in forest and agriculture regions [5], which correspond to the habitat of the Simanjiro district. Our case suggests that the presence of the vector has eventually led to autochthonous VL in Tanzania.

The negative IT Leish rapid test from the peripheral blood and the bone marrow aspirate led to initial uncertainty in the interpretation of the bone marrow aspiration smear. As reported from a Kenyan population, this test has a sensitivity of 89.3% and specificity of 89.8% [6] and could have led to misdiagnosis in the presented case. This fact emphasizes the need for a more sensitive rapid test in East Africa as the authors of the aforementioned publication from Kenya [6] concluded.

The described skin lesions of the patient could be interpreted as post-kala-azar dermal leishmaniasis (PKDL), but no further diagnostic procedure confirmed this. However, the concurrent appearance of VL and PKDL at the time of diagnosis is described in Sudan with a mean time to occur of 0.5 to 13 months after VL and affects mainly younger children [7]. Also, delayed diagnosis and a presumably disease course of more than 3 years might have contributed to this clinical picture in our patient. PKDL in East Africa is often self-limiting and occurs in approximately 50% of cases [7].

The main symptom that has led the patient to seek medical attention was chronic diarrhea. The Ministry of health from Sudan describes in the “Manual for the Diagnosis and Treatment of Leishmaniasis”, that diarrhea is present in half of the Sudanese cases [8], but otherwise rare if the patient is immunocompetent [9].

In our case, the disease was in a disseminated stage and the patient was already cachexic and massively impaired due to very late diagnosis of the disease that would have been diagnosed earlier in a known endemic region.

### Ethics statement

Consent form for case presentation was signed by the patient, and consent form for the use of postmortem findings for the case report was separately signed by the father.

### Key learning points

- Visceral leishmaniasis (VL) is present in Northern Tanzania and must also be considered in patients without travel history to known endemic areas, whereby the clinical presentation can differ from majority of patients due to delay in diagnosis in Tanzania.
- Splenomegaly, prolonged irregular fever, weight loss, and pancytopenia should urge clinicians in Tanzania to include VL in their differential diagnosis.
- Diagnosis can be established by identification of parasites in Giemsa-stained smears of splenic, bone marrow, or lymph node aspiration biopsy [10]. Splenic aspiration is the most sensitive technique.
- The sensitivity of rapid tests misses 10% of positive cases in East Africa. Microscopic and/or PCR diagnostics is advisable in patients with clinically suspected VL and negative rapid test.
- Liposomal Amphotericin B IV is the treatment of choice for VL [10].
